# Cognitive Training in Parkinson's Disease: A Review of Studies from 2000 to 2014

**DOI:** 10.1155/2016/9291713

**Published:** 2016-09-05

**Authors:** Daniel Glizer, Penny A. MacDonald

**Affiliations:** ^1^MacDonald Lab, Brain and Mind Institute, University of Western Ontario, London, ON, Canada; ^2^Clinical Neurological Sciences, Schulich School of Medicine and Dentistry, University of Western Ontario, London, ON, Canada

## Abstract

Cognitive deficits are prevalent among patients with Parkinson's disease (PD), in both early and late stages of the disease. These deficits are associated with lower quality of life, loss of independence, and institutionalization. To date, there is no effective pharmacological treatment for the range of cognitive impairments presented in PD. Cognitive training (CT) has been explored as an alternative approach to remediating cognition in PD. In this review we present a detailed summary of 13 studies of CT that have been conducted between 2000 and 2014 and a critical examination of the evidence for the effectiveness and applicability of CT in PD. Although the evidence shows that CT leads to short-term, moderate improvements in some cognitive functions, methodological inconsistencies weaken these results. We discuss several key limitations of the literature to date, propose methods of addressing these questions, and outline the future directions that studies of CT in PD should pursue. Studies need to provide more detail about the cognitive profile of participants, include larger sample sizes, be hypothesis driven, and be clearer about the training interventions and the outcome measures.

## 1. Introduction

Parkinson's disease (PD) is a disorder characterized by degeneration of dopamine-producing cells in the substantia nigra (SN) and to a much lesser degree in the ventral tegmental area (VTA). This deficiency produces the cardinal motor symptoms of tremor, rigidity, and bradykinesia [1]. Additionally, cognitive symptoms are now also recognized as an undisputable feature of PD [2, 3].

The pathophysiology of cognitive deficits in PD is complex. It seems likely that at least some cognitive deficits result from striatal dopamine deficiency [4–6]. Dopaminergic drugs used to treat motor symptoms in PD have also been implicated in diverse cognitive deficits, proposed to be due to an overdosing of the relatively spared VTA [7–12]. In addition to dopaminergic pathways, dysregulation in cholinergic [13, 14], serotoninergic [15, 16], and noradrenergic [17–19] pathways potentially contributes to cognitive deficits in PD. Alpha-synuclein-containing Lewy bodies deposited in SN and cortex have also been strongly associated with the development of dementia in PD in early and especially at later stages of the disease [20–22].

Although motor impairments are well addressed by dopaminergic medications and deep brain stimulation [23, 24], cognitive symptoms, perhaps due to their complexity and variability from patient to patient, lack clearly effective therapies. Dopaminergic medications improve some cognitive functions but worsen others [7, 10, 23–26]. Further, the clinical significance of these effects has not been systematically studied in placebo-controlled randomized trials. Finally, cholinesterase inhibitors improve cognition and quality of life (QOL; for review, see [27]) but these therapies are limited to patients who are diagnosed with clinically significant dementia and not lesser cognitive impairment. Additionally, the effects on cognitive dysfunction are minimal, not sustained with advancing disease, or sufficient to produce truly meaningful enhancements of function [28]. In sum, neither dopaminergic treatments nor cholinesterase inhibitors modify disease progression, being merely prescribed for symptomatic relief. Investigating effective nonpharmacological treatment options for cognitive decline in lieu of or to enhance available pharmacological treatment in PD is therefore an important area of research.

To better understand what treatments might be useful for cognitive decline in PD, there is a need to better characterize the cognitive impairments associated with this disease. Cognitive decline in PD includes impairments in diverse functions and skills. To date, there is considerable evidence of impairments in executive functions such as working memory (WM), attention, reasoning, and planning even in early, nondemented PD patients [29–32]. Additionally, more basic perceptual visuospatial and verbal processes have been shown consistently to be impaired in PD [32–35]. Impairments in memory have also been documented [36].

Cognitive deficits are very prevalent in PD patients. Even at the time of PD diagnosis, approximately 30–50% of patients already exhibit symptoms consistent with mild cognitive impairment (MCI) or dementia [37] and from 60 to 80% of cases develop into full dementia within 10 years [38, 39]. Cognitive impairments are strongly related to lower QOL ratings and challenges in activities of daily living in patients [40–43]. These deficits present challenges to everyday functioning [41, 44] and are a major cause of loss of independence and institutionalization in PD [45]. Consequently, effective therapies for cognitive impairment in PD are an important but unmet need [23, 45]. Exploration and empirical testing of interventions to address cognitive decline in PD are imperative.

Over the last decade, nonpharmacological treatments that aim to improve cognition have increasingly been a focus in healthy aging as well as in various clinical populations other than PD. Cognitive training (CT), a nonpharmacological intervention, has generated significant interest and engendered a wealth of research. CT is an approach that broadly encompasses the idea that repeated performance of cognitive tasks leads to strategy development or brain changes that improve cognitive functions either within a specific domain or in general.

In healthy populations, evidence suggests that CT can benefit older adults through either restorative or protective factors [46–51], although other studies appear to find modest or no effects following CT [52, 53]. Thus, it remains to be seen whether a CT intervention can be developed that leads to meaningful effects in a healthy population. A thorough review of this controversy, however, is beyond the scope of the current review, which will focus more on CT in PD.

*CT in Clinical Populations*. In contrast to studies in healthy controls, in clinical populations, CT has shown much more promising and consistent results. CT and attention training have been found to improve visuospatial and language abilities in patients with aphasia and neglect syndromes following traumatic brain injury (TBI; [54, 55]). Several reviews and meta-analyses of TBI treatments concluded that CT approaches have potential as remediation strategies after stroke but noted that further research is warranted [56, 57]. In conjunction with other approaches, CT has been successfully employed in the treatment of disorders such as schizophrenia [58–60], Attention Deficit Hyperactivity Disorder (ADHD), and various addictions and mood disorders [61–64]. Finally, in at-risk populations, such as older adults susceptible to Alzheimer's disease and dementia, various forms of CT show protective effects and even improvements in select cognitive functions [49, 51, 65–67].

There have now been a number of studies investigating the effect of CT on cognitive dysfunction in PD. In this review, we present and summarize each study individually, discuss the potential of CT as a therapy for cognitive impairment in PD, highlight knowledge gaps, and make recommendations for future studies. We will critically evaluate the design and methods of studies of CT in PD. The ultimate goal of this review is to focus the research on CT in PD, to suggest guidelines for future studies, and to highlight common issues that are noted in the literature.

*Literature Search*. To identify all studies that investigated CT to treat cognitive symptoms of PD, we conducted a search in PubMed and PsycINFO using the following key terms and combinations: cognit^*∗*^ train^*∗*^ AND Parkinson^*∗*^; memory train^*∗*^ AND Parkinson^*∗*^; attention train^*∗*^ AND Parkinson^*∗*^; cognit^*∗*^ rehabilitation AND Parkinson^*∗*^; memory rehabilitation AND Parkinson^*∗*^; attention rehabilitation AND Parkinson^*∗*^; cognit^*∗*^ remediation AND Parkinson^*∗*^; memory remediation AND Parkinson^*∗*^; attention remediation AND Parkinson^*∗*^. We selected for further inspection studies that included information on (1) the training group(s), (2) the training intervention, (3) the outcome measures, and (4) specifically used CT interventions, alone or in combination with (an)other nonpharmacological therapy in PD. We found only 13 studies that met all of these criteria. In each study, we examined whether (1) there was a control group or comparison intervention, (2) training was multimodal, computerized, or pen and paper, (3) CT was combined with another intervention, (4) CT was standardized or individually tailored, and (5) QOL changes were assessed.  Table 1 lists the identified studies and categorizes them according to design.

## 2. Results

### 2.1. Single Group, Uncontrolled Studies

In a small preliminary study of CT with inpatients, Sinforiani et al. [68] examined the effects of a rehabilitation program consisting of motor and cognitive training in patients with early stages of PD and mild cognitive decline but not dementia. They used Training Neuropsicologico (TNP; [82])—a computerized CT program aimed at improving attention, abstract reasoning, and visuospatial abilities. The PD patients (*N* = 20) who enrolled in the program for 12 sessions showed significant improvement on measures of verbal processing and verbal memory as well as on one measure of abstract reasoning. These improvements remained when examined at a six-month follow-up. However, without a control group, it is impossible to attribute improvement to CT specifically. Alternatively, change in function could have owed to nonspecific effects of being enrolled and followed in a study, to the passage of time, to fluctuations in clinical disease with regression to mean behaviour, or to decreased anxiety or stress of performing with repeated exposure to the setting. Additionally, participants were an inpatient group at a rehabilitation centre, making them a special subset of the PD population. This scenario enhances the possibility that improvement represented regression to mean performance, with the passage of time of the inciting event or circumstance leading to admission to a rehab setting and later testing. Other confounds exist due to the rehabilitation setting. The group received motor rehabilitation in addition to the CT program so the influence of the two cannot be teased apart. Additionally, it is important to note that* a majority of the measures of cognition yielded no significant improvement, including measures of overall cognition (Mini-Mental State Examination score, MMSE), WM (digit span, Corsi block), and measures of cognitive flexibility (Wisconsin Card Sorting Test, WCST),* and the authors did not indicate if corrections for multiple comparisons were applied.

In another study, Mohlman et al. [69] examined the acceptability and feasibility of administering CT to patients with PD. Participants (*N* = 16) completed neuropsychological tests and psychometric questionnaires before and after training to assess changes in cognition and mood. The neuropsychological battery consisted of the digit span forward and backward tasks, the Stroop Color Word Test, the Trail Making Test (TMT), and the Controlled Oral Word Association Test (COWAT). The psychometric tests included the Penn State Worry Questionnaire, the Beck Anxiety Inventory (BAI), the trait scale of the State-Trait Anxiety Inventory (STAI), and the Beck Depression Inventory (BDI). During the training period, which lasted one month, with 90 minutes of training per week, participants came to the lab on university campus and performed the Attention Process Training (APT-II) Intervention, a computerized CT program. The modules included in the APT-II focused on training sustained attention, selective attention, alternating attention, and divided attention. Participants also received daily homework assignments throughout the month. The main focus of the study was to determine the acceptability and feasibility of the CT across 4 dimensions: fatigue, effort, progress, and enjoyment. Findings indicated that participants showed good acceptance and completion of the training program. In addition,* all participants' scores on the 4 neuropsychological tasks improved from pre- to postintervention (though no statistics were provided)*. As the main focus was on acceptability of the CT, the article did not include information about the cognitive performance of the group before or after training.

A study by Disbrow and colleagues [70] investigated the effects of executive function and motor focused CT on performance of a similar motor sequence learning task, as well as measures of cognitive flexibility, verbal fluency, and timed instrumental activities of daily living (TIADL). They enrolled 30 PD patients and 21 age matched controls. During pretraining, participants performed a motor sequence learning task (which also served as the training task), the TMT, the Delis-Kaplan Executive Function Scale (D-KEFS), and the Timed-Up-and-Go Test (TUG). In the motor sequence task, participants had to press on a keypad the sequence of numbers corresponding to the sequence that was displayed on the screen (e.g., 1-3-4). Sequence length varied between 1 and 4 digits and included two conditions, the Externally Cued (EC) condition, where feedback was displayed on the screen every trial, and an Internally Represented (IR) condition, where feedback was not displayed on the screen every trial. Based on performance on the motor sequence task, PD patients were split into two groups for further analyses, an impaired performance group (*N* = 14) and an unimpaired performance group (*N* = 16). Outcome measures for the motor sequence learning task were time to initiate motor response, time to end sequence, and number of errors. During the training period, participants performed the motor sequence task for 10 sessions each taking 40 minutes, over the course of about two weeks.

Results showed that training benefitted both the EC and the IR conditions in all groups. Although after training the impaired PD patients still had slower initiation and completion times in the EC condition than the unimpaired PD patients and controls, their performance in the IR condition improved after training and was not significantly different from the other two groups. This indicates that the training improved motor performance dependent on executive function, as required when participants internally represent and plan a sequence but not a simpler version of motor performance when feedback and digit sequence are shown. The previously impaired PD patients also made fewer errors after completing training, similar to the unimpaired PD group and the control group. Training did not have an effect on the D-KEFS, the TIADL, or the TUG. There was a main effect on training on the TMT B minus A scores; however, the impaired PD patients still showed impaired performance after the training compared to the other two groups.

*Overall, the results of this study suggest that patients with specific impairments can particularly benefit from specialized, focused training. It is important to note that the training and outcome tasks were nearly identical; thus, it is unclear whether the effects of this type of training transfer to other functions*. Improvement on the TMT suggests that there may be some degree of transfer although no effects were found on measures of QOL and other motor tasks. Moreover, there was no waitlist PD group so it is impossible to attribute improvement solely to the training rather than repeated testing or the passage of time.

### 2.2. Waitlist-Controlled Studies

In a study employing neuroimaging to investigate CT, Nombela et al. [71] scanned participants using fMRI before and after training. Ten participants with PD and ten healthy age-matched controls completed a variation of the Stroop task at baseline and after training. Half of the PD patients were enrolled in a training intervention (*N* = 5), and half served as the untrained waitlist control group (*N* = 5). Training consisted of a series of Sudoku puzzles completed at home every day for the duration of six months, with weekly meetings with the researchers to go over the puzzles. During baseline assessment, participants completed an easy Sudoku puzzle, the modified Stroop task, and several questionnaires evaluating cognition and PD symptoms (MMSE, Unified Parkinson's Disease Rating Scale (UPDRS), Montgomery-Asberg Rating Scale). PD patients had slower response times (RTs) on the Stroop task, more missed trials, and poorer performance overall. They also took longer to complete the easy Sudoku puzzle compared to controls. Functional neuroimaging revealed more extensive brain activation in patients than in controls and less activation in the left precentral gyrus, left medial frontal gyrus, right precuneus, and the left inferior parietal gyrus. After the six-month training period, the trained PD group had faster RTs on the Stroop task, more correct answers, and fewer missed trials than the untrained patients. Their RTs and correct and missed trials were also better than during their baseline assessment. Further, they completed the Sudoku puzzles more quickly than the untrained PD group. The brain activation of the trained PD group during the Stroop task was more similar to that of the healthy control group. The results of this study suggest that daily performance of cognitive exercises can improve performance on these exercises as well as other related cognitive tasks, but the study is limited by a small sample size and a very unusual and time consuming intervention. Additionally, the assignment to the training group was not random but voluntary, leading possibly to fundamental differences between the training and untrained groups, with the former being more engaged and enthusiastic participants.

A study by Naismith et al. [72] combined psychoeducation and CT and found that, compared to a waitlist control group (*N* = 15), the treatment group (*N* = 35) improved on measures of learning and memory retention. The CT intervention was based on the Neuropsychological Educational Approach to Remediation (NEAR), was individualized to each participant, and comprised a wide array of commercially available computer-based programs depending on the individual's strengths and weaknesses. Participants completed 14 training sessions over two weeks in a lab group-setting. The primary outcome variable was episodic memory measured through the Logical Memory subtest of the Wechsler Memory Scale III (LOGMEM). Secondary measures consisted of psychomotor speed and mental flexibility (TMT), verbal fluency (COWAT), general cognition (MMSE, National Adult Reading Test), and knowledge about PD assessed using a multiple choice questionnaire. Results revealed that the CT group improved more than the waitlist group on LOGMEM (learning and memory retention). There was no improvement on measures of psychomotor speed, mental flexibility, verbal fluency, or depressive symptoms. The results lend support to CT as a viable intervention to possibly slow down memory decline in PD patients and improve performance on some memory and learning tasks. Due to the difficulty of administering such a comprehensive and individually tailored intervention as well as the high degree of variability in terms of the intervention between patients, it is difficult to assert whether these effects might generalize to PD patients broadly.

A randomized, waitlist-controlled study by Edwards and colleagues [73] investigated the effect of a processing speed training intervention on useful field of view (UFOV), self-rated cognition, and depressive symptoms. One group of PD patients received Speed of Processing Training (SOPT; *N* = 44), and a second group of PD patients served as a waitlist control (*N* = 43). The groups did not differ on any motor, cognitive, or demographic measures at pretraining. The training intervention consisted of a SOPT program (InSight, Posit Science, Inc., San Francisco, CA) which included five exercises focusing on rapid processing of visual stimuli, selective attention, and visual working memory. Training was self-administered, computerized, and completed at home. The intervention lasted for three months, with a recommended schedule of three sessions per week, each session taking an hour. Outcome measures were UFOV, the Cognitive Self-Report Questionnaire, and the Centre for Epidemiological Studies Depressive Symptoms Scale (CES-D).* Analyses revealed that although both the SOPT and the waitlist group showed significantly improved performance on the UFOV task, the SOPT group improved significantly more from pre- to posttraining than the waitlist group. The other two measures, self-reported cognition and depressive symptoms, did not show any changes*. The results of this study provide evidence that SOPT, even when self-administered and completed at home, can lead to improvement in similar tasks, more than can be accounted for by test-retest effects. An important caveat the authors mention is that the effects were most strongly associated with factors accounting for less severe PD stage (e.g., age at onset, disease duration, and L-dopa equivalent dosage). Additionally, none of the patients had symptoms consistent with MCI; thus it will be helpful to conduct a similar study with MCI patients to evaluate whether the SOPT program can benefit more severe stages of PD or cognitive decline.

### 2.3. Studies Comparing CT to Standard Treatments

Sammer et al. [74] investigated the effectiveness of CT with inpatients at a rehabilitation centre. Participants were divided into two groups: one group (*N* = 12) received a treatment focusing on executive functions; the other group (*N* = 14) completed a standard treatment comprised of occupational therapy, physiotherapy, and physical treatment sessions. The executive function intervention consisted of a range of both standardized and novel tasks training WM, abstract reasoning, problem solving, visuospatial processing, and verbal processing. After 10 training sessions over the course of a 3-4-week hospital stay, only the executive function treatment group improved on some measures of executive function and WM (Behavioural Assessment of the Dysexecutive Syndrome-rule shift and 6-element subtests). However, there were other measures of WM and executive function (TMT and a face-name learning task) and a measure of attention, on which neither group improved. There was also no change in ratings of well-being or depression between the two groups. The results of this study provide some limited evidence that CT can lead to enhancements of executive function. However, it is necessary to identify why some tasks of executive function showed improvement whereas other tasks of executive function did not. With no corrections for multiple comparisons, it is possible to find statistically significant differences in a subset of many tasks due to chance alone, which cannot be ruled out in this case.

In a randomized, controlled, experimenter-blinded study of CT in patients with PD, París and colleagues [75] compared the effects of an intensive individualized CT program (*N* = 12) to a speech therapy intervention (*N* = 12). Each participant in the CT group received individual training using a platform of 28 tasks (i.e., SmartBrain computerized program) focusing on specific cognitive domains known to be impaired in PD patients such as memory, attention, WM, executive functions, visuospatial abilities, and psychomotor speed. They also trained on nonspecific tasks that tapped overall cognition including language, simple calculations, and culture. Additionally, participants received homework exercises to be completed outside the sessions. The speech therapy participants received group-sessions focusing on communication difficulties as a result of PD. The intervention program for both CT and speech therapy groups consisted of 12 sessions over four weeks, each session lasting 45 minutes. The CT group also received 20 weekly homework exercises to stimulate cognition. At a baseline assessment, participants completed a comprehensive battery of tasks measuring overall cognition (e.g., MMSE), attention and WM (e.g., digit span), information processing speed (e.g., TMT), verbal and visual processing, learning, and executive functions (e.g., Tower of London (TOL), Stroop test), as well as questionnaires assessing QOL and mood.* Following the training period, the CT group showed significantly more improvement than the speech therapy group on measures of attention, processing speed, memory, visuospatial abilities, executive function, and semantic and verbal fluency. There was no difference between the groups on measures of QOL or mood*. More importantly, although many outcome measures were included, not all measures showed improvement, and there was no indication that analyses were corrected for multiple comparisons. Despite describing aspects of cognition that the training program focused on, the specifics of each training task were not included in the manuscript, thwarting wide-spread implementation. These details are also needed to determine how well the trained skills transferred to the outcome measures, and whether the training effect generalized to similar or diverse tasks.

In a study examining the effects of a CT-like intervention on symptoms of PD and independent activities of daily living measured by the UPDRS-II, Pompeu and colleagues [76] divided 32 PD patients into two groups. Both groups received an intervention consisting of 14 sessions of 30 minutes of global physical exercises. The control group (*N* = 16) received additional 30 minutes of balance exercises, whereas the training group (*N* = 16) received 30 minutes of training using WiiFit games. WiiFit games focus on motor performance (e.g., Torso twist, soccer heading, basic step, and speed run), though cognitive processes such as planning, decision making, and divided attention are invoked to perform the tasks. The main outcome measure,* performance of activities of daily living as assessed by the self-report on the UPDRS-II,* revealed* no difference between the two groups before training, after training, or at 60-day follow-up* evaluations. Both groups indicated improvement on the UPDRS-II, leading the authors to conclude that training using the WiiFit games does not lead to any improvement over performance of general balance exercises. However, the WiiFit games are designed primarily to focus on motor performance rather than cognitive processes. It is likely that the chosen WiiFit games did not have a clear focus on any aspect of cognition* per se* and instead the cognitive training occurred as a by-product of performing the motor task. Although the authors claim that the WiiFit games trained cognition, the CT tasks and the cognitive evaluations were a secondary measure and were not clearly defined.

Another study conducted by Peña et al. [77] compared a structured, pen and paper CT program to occupational activities. Outcome measures were processing speed (TMT-A, Salthouse Letter Comparison Test), verbal learning and memory (Hopkins Verbal Learning Test), visual learning and memory (Brief Visual Memory Test), executive function (Stroop), and Theory of Mind (Happé test). The CT group (*N* = 22) received a standardized intervention (REHACOP, a Spanish cognitive rehabilitation program for psychosis) focused on improving attention, memory, language, verbal processing, executive function, and theory of mind, as well as general cognition and functional disability ratings. The occupational therapy group (*N* = 22) performed activities such as drawing, reading the newspaper, and arts and crafts. Both groups completed 39 sessions over 13 weeks, three per week, with each session taking an hour. They found that, following training, the* CT group showed more improvement than the occupational therapy group on measures of processing speed, visual memory, theory of mind, and functional disability*. This provides further evidence that structured CT is more beneficial than interventions not explicitly focused on cognitive improvement. Also, the improvement on the functional disability scale suggests that CT might lead to benefits that generalize to functional activities. However, the training program is quite a bit longer than those usually studied in CT so results are difficult to compare to other studies.

A study by Cerasa and colleagues [78] compared a computerized CT program designed to rehabilitate attention in patients with multiple sclerosis (REHACOM) to a group performing a simple visuomotor coordination tapping task. Participants were also scanned using fMRI at resting state before and after training. Both the CT group (*N* = 8) and the PD control group (*N* = 7) completed 12, one-hour training sessions over six weeks. On the outcome measures, which included a range of tasks assessing verbal memory, spatial memory, verbal fluency, information processing speed, visuospatial processing, mood, cognition, and QOL, the CT group improved more than the control group on two tests. In some cases improvement was found in only some tests but not others from the same cognitive domain, or even tests that are similar to each other (e.g., digit span forward improved whereas digit span backward did not). There was also a difference in resting state brain activity in the left dorsolateral prefrontal cortex within the left central executive network between the CT and the PD control group. Overall, results from the study provide weak evidence that CT can lead to improvements in cognition and some changes in brain activation. However, no differences between the groups were found on most cognitive measures, and also on measures of QOL and mood, so these effects do not seem to benefit daily functioning.

### 2.4. Studies Comparing Different Forms of CT

Reuter et al. [79] conducted a large scale study of CT with inpatients and their caregivers, examining the effects of three intervention programs on tests of memory, language, reasoning, attention, executive function, and visuospatial processing, measured with the Alzheimer Disease Assessment Scale-Cognition (ADAS-Cog) and the Scale for Outcomes in Parkinson's Disease-Cognition (SCOPA-Cog) testing batteries. Measures of general cognitive function (Parkinson Neuropsychometric Dementia Assessment (PANDA) and MMSE), QOL, and activities of daily living (Parkinson's disease questionnaire (PDQ-39)) were also taken to assess the overall impact of the training programs on cognition. Patients completed the training while staying at a hospital for four weeks, for a total of at least 14 training sessions, and were assessed before, after, and at six-month follow-up.

Group “A” (*N* = 71) completed an array of individually tailored tasks focused generally on executive functions, memory, reasoning, WM, attention and concentration, and planning (for a list of tasks please see Table 2). Group “B” (*N* = 75) received the same program as well as transfer training that aimed to improve management of activities of daily living and increase self-confidence through the use of strategies such as mnemonics, decision making, handling of money, reading comprehension, and other tasks that patients identified as challenging. Group “C” (*N* = 76) received the CT, the transfer training, and motor training, which consisted of games and tasks that focus on inhibitory control, coordination, speed, perception, orientation, WM, attention, and visuospatial abilities. The caregivers of participants from each group also received educational sessions pertaining to the skills practiced with the patients.

*All groups showed improvement on the outcome measures; however, Group C, the group receiving all interventions, showed significantly more improvement than Group A or B across all measures*. Participants in each group also showed* increases in rated QOL*, with Group C reporting the most improvement. At the six-month follow-up, a larger proportion of participants in Group C had retained their skills and improved performance compared to Groups A and B. The results strongly suggest that multimodal rehabilitation programs can lead to significant improvements across a variety of cognitive functions, and that carefully designed, individualized CT programs can generalize to improvement on untrained but similar cognitive tasks. However, there are limitations to such an approach. First, it is difficult to understand and clearly attribute benefits to individual components of the intervention given that all groups received multiple components of active treatment. Such an intervention is very time and resource consuming, because training programs have to be tailored to each participant and therefore widespread application seems unfeasible. Additionally, it requires a significant time commitment from the patients who complete the training program, ranging from four hours per week with a trained professional for Group A, and upward of six hours per week for participants in Groups B and C, which showed the most change.

In a randomized controlled study Petrelli and colleagues [80] examined the effects of a structured and an unstructured CT intervention relative to a waitlist control group on measures of memory, attention, and executive functions, as well as QOL and mood. One group received a structured CT program (*N* = 22) administered using the NeuroVitalis software. A second group received an unstructured CT program (*N* = 22) administered using the MentallyFit program. Finally, a third group was a waitlist control (*N* = 21). Training sessions were completed in a group setting led by a supervisor, and training lasted 12 sessions which took 90 minutes each. At pre- and posttraining evaluations, participants completed a comprehensive battery of cognitive tests and neurological assessments. Primary outcome measures were performance on the Brief Test of Attention (BTA), DemTect, a cognitive screening tool, and Memo, a verbal processing test. Secondary measures included visuoconstruction (Complex Figure Test), depression scores (BDI), and QOL (PDQ-39).

When compared to the waitlist control, the group receiving the structured CT program showed improvement in measures of WM and short term memory, whereas the unstructured CT group showed trends in improvement on verbal memory and fluency. The* unstructured CT group also showed a decrease in depression scores*. The structured CT group showed significantly more improvement than either group on WM measures, as well as a trend in verbal short term memory. This study supports CT as an intervention that can improve performance on untrained measures of cognition and suggests that a structured program leads to more benefits than an unstructured one. The use of many outcome measures that overlap in domains and the fact that some WM tasks showed improvement whereas others did not, weakens the conclusions drawn somewhat. Additionally, the training interventions included various tasks completed in group sessions which reduces the specificity of the intervention and limits the accessibility and independent performance of the CT regimen.

In a controlled, randomized, participant-blinded study, Zimmermann and colleagues [81] compared the effects of a structured CT program and an alternative, nonspecific treatment intervention, on measures of attention, executive function, visuoconstruction, and episodic memory. The CT group (*N* = 19) performed a series of training tasks on the computer using the CogniPlus software. The alternative treatment group (*N* = 20) played an interactive videogame which involved physical activity (WiiSports). Both training interventions ran for 12 sessions over the course of four weeks, each session taking 40 minutes and supervised by a psychologist or trained student, who were not blinded to group allocation. Neuropsychological assessment included parts of the Tests of Attentional Performance battery (alertness, working memory), the TMT, the Block Design Test from the Wechsler Intelligence Scale for Adults, and the California Verbal Learning Test. The alternative treatment group that completed training using the WiiSports games showed significant improvement on the alertness portion of the Tests of Attention relative to the CT group and a trend level improvement on tests of visuoconstruction and episodic memory. These results suggest that a* nonspecific training intervention might be as effective as a CT intervention in improving attention*. However, it is likely that the WiiSports tasks were more novel and engaging than the standardized CT program delivered using CogniPlus, which could explain the improvement in attention. Finally, as the authors note, there is increasing evidence that physical activity promotes cognition [83, 84], potentially accounting for these findings because performance of WiiSports games involves physical activity.

A summary of the studies discussed above is presented in Table 2. Due to largely varied methodologies and relatively small sample sizes it remains unclear whether CT is effective as a wide-spread, cognitive intervention in PD. Reviews of earlier CT studies noted similar limitations [85, 86]. Based on the research published to date, there is insufficient information to determine which training program or schedule is most likely to promote improvements, what outcome measures best estimate the impact of CT, and which cognitive functions benefit most from training.

Due to lack of standardized training programs in this field, there was little consistency or convergence between training tasks or outcome measures, making cross-experimental comparisons difficult and eliminating the opportunity for true replication. Moreover, some studies found improvement across a wide array of tasks and cognitive skills, whereas others found more modest and domain-specific effects. Even when there was improvement on outcome measures, it was seldom explained from a theoretical perspective by the cognitive elements that were targeted by the training regimen. Further, training did not often generalize to other untrained aspects of cognition.

More recent studies of CT in PD used active control groups and compared different CT interventions to one another, as well as to alternative interventions such as psychoeducation, physiotherapy, skill transfer training, and videogames [72, 76–81]. However, there is still crucial information lacking that would enable predictions to the larger PD population or permit widespread and faithful application beyond the study. Studies need to (1) be clear about exact details of the intervention applied to the training group, (2) include larger sample sizes, (3) describe more fully the patient population characteristics in case only subgroups are expected to benefit, and (4) examine effects on QOL and long term outcomes. Providing detailed information about the methodology and task administration will enable comparisons of results across studies.

It is clear that there is burgeoning interest in CT as an intervention in PD, yet due to lack of methodological consistency even the positive results are difficult to evaluate across studies. This problem appears to permeate all areas of research of CT, in healthy younger and older populations, as well as in studies with clinical patients [57, 87]. Several reviews still note that methodological limitations are holding the field back [66, 87, 88], and these need to be addressed so that CT can be examined with the scientific rigor and standardized protocol that many pharmaceutical and behavioural interventions currently undergo. In effect, there are no clear replications and consequently the legitimacy of CT as a therapy for cognitive impairment in PD has not been conclusively determined. This is in line with a recent meta-analysis suggesting that the evidence for CT in PD is not robust and more research is needed [88]. In the discussion that follows we examine several of these issues in more depth and provide suggestions for unifying the research in this field.

## 3. Discussion

### 3.1. Cognitive and Demographic Profile of Participants and Implications for CT Effects

If investigations of CT in PD hope to address the ambiguity regarding training effects and the extent to which training can benefit individuals, there is a need to consider the cognitive and demographic profile of the studied sample. Demographic and clinical characterization of participants in future studies should more clearly describe the groups under study as these patient features might interact with CT effects. This will also define the groups to which findings might be applicable because PD patients can vary vastly in their cognitive aptitudes depending upon stage of disease, and some interventions might be more suitable to relatively unimpaired patients, whereas others could be particularly beneficial for patients showing more severe decline. Therefore, studies need to clearly describe the severity of disease and provide measures indicating the extent of cognitive decline, both as an overall score and ideally as a composite of different cognitive domains as recommended by the MDS Task Force [89]. It is also necessary to consider the effect different disease severity (as measured by the Hoehn and Yahr scale or the UPDRS) can have on the ability to complete the training intervention either autonomously or with assistance, and how this might impact performance on outcome measures.

Many studies of CT in PD exclude patients with dementia or MCI, enrolling only PD patients who are clinically cognitively intact. Considering that baseline cognitive function is a variable that will likely strongly impact CT effects, full characterization of PD patients included in studies needs to be disclosed. Finally, studies that explicitly contrast PD groups, formed on the basis of cognitive abilities, are needed to directly investigate this issue though only one has been conducted to date [70]. Of the studies reviewed, some included participants with MCI and others included only cognitively healthy patients (see Table 2). Since the effects of CT are likely different for cognitively healthy versus cognitively impaired participants, it is impossible to make conclusions about the effectiveness of CT when one study employs a cognitively healthy population and another employs patients with MCI. The interpretation of the results is limited further when the participants are not thoroughly defined in terms of their cognitive abilities or disease severity.

### 3.2. Mechanisms Underlying CT

Over the last decade several studies found that CT can lead to functional and structural brain changes. Most commonly and reliably, fMRI studies have shown improvement-correlated changes in activation in frontostriatal networks, the dorsolateral prefrontal cortex (dlPFC), medial PFC (mPFC), and the parietal cortex (PC) following CT [61, 71, 90–94]. Functional connectivity (FC) analyses have revealed increased connectivity following CT in areas of the PFC, PC, and the basal ganglia [95, 96]. Studies have also observed functional changes using measures of cerebral blood flow (CBF) in the Default Mode Network (DMN) and the External Attention System, as well as globally [96, 97].

Recently, Chapman and colleagues [97] observed both functional and structural changes in healthy seniors following CT. The authors found increased global and regional CBF in the DMN and the central executive network as well as greater connectivity in these regions, compared to a waitlist group. They also found differences suggesting changes in white matter integrity, which could be due to increased axonal myelination. More support for structural changes comes from McNab and colleagues [98], who used Positron Emission Tomography (PET) and found changes in dopamine D1 receptor density and binding potentials in the PFC and PC after 14 hours (across five weeks) of training. These changes were correlated with behavioural improvement in WM tasks. Finally, in nonhuman primates, WM training has been shown to lead to changes in neuronal firing patterns, leading to the recruitment of more neurons but a less variable and correlated firing rate (for review, please see [99]).

These findings that CT leads to brain changes and potentially normalization of activation and connectivity patterns are intriguing and increasing the plausibility of CT as an effective therapy (see review in [91]). However, more research is needed to understand the nature of these changes. There is as of yet no consensus that these changes reflect actual restorative processes of impaired brain function/structure integrity in clinical populations. An alternative explnatio is that brain changes could reflect protection from cognitive decline given that these alterations occur in healthy older adults performing CT who show less decline than a waitlist comparison group [97, 100]. The changes in brain activation and structure notwithstanding, at a behavioural level, CT likely imparts consciously and/or unconsciously developing cognitive strategies that permit more effective task performance. One such example could be the use of mnemonics or other memory aids, as well as chunking of items to reduce memory load (e.g., as in [93]). Ultimately, whatever the mechanism, whether due to neural alterations or acquisition of new, more effective cognitive strategies, it remains unclear whether these alterations are long lasting or temporary, and whether they correlate with improvement in daily tasks.

### 3.3. Selecting and Characterizing Outcome Measures of CT

Before CT can be established as a therapeutic or preventative measure of cognitive dysfunction in PD, it is necessary to demonstrate that completion of a CT program translates into improvements in untrained contexts and activities. To evaluate the effectiveness of CT, there needs to be some indication that general skills or functions improve* and *that this improvement transfers to other untrained activities. Discussing CT-mediated changes with reference to learning and transfer of learning literatures (e.g., [91, 101, 102]), training on one task should, at a minimum, lead to improvements in similar tasks that invoke the same cognitive processes or strategies. This is termed* near transfer*. An example of near transfer would be improvement on an N-back task, requiring WM maintenance and updating, following training on a digit span task, also requiring WM maintenance. Though these are different tasks on the surface, both engage and depend on WM processes. In this way, improvements in one task following training of the other presumably result from general enhancement of WM processes. An ideal CT regimen, however, would not only produce near transfer effects but in fact optimize performance of very different tasks or skills, relying on quite disparate cognitive processes from those that were trained. This is referred to as* far transfer*. An example of far transfer would include practice on a digit span task augmenting efficiency of designing a multistep plan to achieve a goal in the Tower of Hanoi task. Far transfer effects potentially arise due to shared cognitive processes or strengthening of more general cognitive processing. CT-related improvements only on trained tasks that do not translate to benefits outside the specific experimental context, termed* direct transfer* or simply* training effects*, would be trivial, having little importance given the aim of addressing cognitive impairment in PD in the real world. That is, though training effects can have value in some scenarios where skill learning is the focus, for example, in learning to fly a plane, these would be insufficient to merit investment of time or resources for the stated purpose of preventing or remedying cognitive dysfunction in PD. Studies investigating CT effects need to state clearly the degree of transfer effects that they have achieved so their value can be understood.

Although there is some evidence of what might constitute far transfer of skills in PD in some of the studies that were reviewed, these effects are difficult to ascertain because often multiple tasks are included in training interventions without explicit design to test far transfer. In part this relates to the fact that most studies use training paradigms that are unfocussed, incorporating tasks that train many cognitive domains within a single regimen to increase the probability of a successful outcome. While pragmatic, this approach unfortunately makes it very difficult to identify the specific component(s) of the training intervention that promotes improvement. Future studies should employ the concepts of direct, near, and far transfer explicitly in their hypotheses, choice of interventions, and corresponding outcome measures to investigate these issues more clearly and provide a context for the results.

Ultimately, it is important to test whether CT leads to any QOL changes. Studies that have found improvement on these measures delivered CT either in a social group setting or in one-on-one sessions with an instructor (e.g., [69, 76, 77, 79]). In this way, the improvement was potentially confounded by increased social contacts and a greater sense of involvement in a community rather than the specific CT regimen. Although from a practical perspective these improvements are desirable regardless of the underlying cause, from the perspective of gaining theoretical understanding and for evolving recommendations regarding the most effective approaches, the specific effect of a CT regimen on QOL and mood needs to be isolated from other nonspecific effects. To tease apart these influences, it would be necessary to compare the same CT when self-administered versus when it was delivered in a group, attending to QOL changes related to each intervention. Future studies must establish whether CT specifically enhances QOL and performance of daily activities, as these are ultimately the changes that are most important to patients with cognitive impairments. Subjective benefit in real life function is an important endpoint. Many studies to date did not examine the effect of CT-derived improvements in PD on everyday QOL.

### 3.4. Description of Interventions

There is a significant lack of clarity, detail, and consistency regarding CT interventions in PD. No gold-standard CT program has been developed to date; consequently many different CT interventions have been investigated. A variety of tasks tend to be used as part of any given CT regimen. In some studies, the intervention comprised a developed standalone CT program, whereas in others, the intervention consisted of a multitude of training tasks with no overarching theoretical basis for inclusion. Additionally, when including a task as an outcome measure, it should be noted why this task is chosen and what is the expected outcome (e.g., decrease in reaction time, higher accuracy, and fewer steps taken). Interventions and outcome measures tend to be chosen due to convenience and availability, and no true replications have been achieved. There is a dire need for consistency in the literature so that results of different studies can be synthesized and compared in a more meaningful way. The design of future CT studies should be more programmatic and theoretically motivated. Ideally, the training regimen should consider known cognitive impairments in PD. The specificity of the target training regimen should be determined by comparing to a task or set of tasks that train cognitive skills that are not known to be impaired in PD. Finally, outcome measures should be selected to represent broad cognitive function to evaluate near and far transfer effects. Following this more reasoned approach, the probability of deriving CT programs that are effective and impactful seems increased.

A related issue is that some studies individually tailored CT to each participant, whereas others used the same tasks and levels of difficulty for all participants. Although tailored training in theory might be expected to lead to better outcomes, this has not been proven and therefore the time-consuming and costly nature of this approach is not empirically justified yet. To fully explore this, a study would need to directly compare a group receiving a tailored intervention (based on deficits in baseline performance) taken from a battery of standardized tasks, with another equivalent group receiving a random selection from the same battery of tasks. If the patients that received the individualized training benefit more from the intervention than the random training group, there will be merit in adjusting a training program for each participant on an individual basis. We offer that until such a study has been conducted, a middle ground would be selection of tasks and CT programs that take into account the cognitive profile of PD patients. That is, CT would be tailored not to each individual, but to the PD population as a whole. It appears that recent studies do indeed employ such an approach; however, there needs to be stronger theoretical backing for training task and outcome measure selection as described in the preceding paragraph. Finally, studies should attempt to select tasks and programs that have parallel versions to control for test-retest effects between baseline and posttraining. Again, direct transfer or practice effects are of little value given the aim of rehabilitating cognition in PD outside of the experimental context.

One of the challenges of CT programs is that they tend to be time-consuming and generally require the presence of an administrator to lead the session, especially during group sessions. This might limit the accessibility and availability of the CT program for patients who live remotely, mobilize with difficulty, or for other reasons are unable to attend the sessions. Some might simply prefer the convenience of in-home regimens. Computerized CT programs have been developed with these notions in mind and allow participants to complete the program on a variety of electronic devices, including home computers, laptops, and even tablets or phones. Computerized CT is potentially more convenient for some patients, allowing for more accessibility and conferring a feeling of autonomy. On the other hand, some patients might feel daunted by the technology which could be a disadvantage. Studies of computerized CT programs in healthy older adults and individuals with TBI, schizophrenia, and PD show that these computerized programs can be as effective as or even more effective than traditional pen and paper programs [48, 50, 56, 67, 94, 103]. It remains undetermined which approach is more effective in PD, however, without head-to-head comparisons. This is an important empirical question that needs to be resolved given the expense of one-on-one administration of some programs. Once again, a direct comparison of the same CT delivered by an administrator or in a pencil and paper version versus a computerized format is necessary to address this question. Until then, this remains a confounding factor with some studies administering computerized CT whereas others spend face-to-face time with patients to provide training.

Finally, there has been no investigation of the appropriate length of an individual training session or the number of sessions that are needed to produce positive effects. Further, the question of whether promoted changes are enduring remains unanswered. The duration of training courses seems chosen for practical reasons (e.g., the duration of admission to a rehabilitation center) or at random with virtually no justification for the parameters that were chosen. Going forward, investigating dose effects, by varying and comparing effects of more or less intense and prolonged CT regimens, will be needed.

### 3.5. Replication and Multiple Comparisons

Despite the many comparisons conducted in each CT study, there is seldom a statistical adjustment for multiple comparisons. This greatly weakens our confidence in the results, as performing a large number of comparisons will inflate the chance of finding differences in pre-post intervention measures or across comparison groups due to chance alone. This confidence would be increased if on an* a priori* basis a chosen regimen was predicted to improve some skills relative to others. Further, greater confidence would be inspired by similar effects of CT on outcome measures that gage the same cognitive domain. In our review, we often found inconsistent effects of CT on measures tapping into a common cognitive domain, though more often studies were simply not designed to allow for this conceptual replication. Most studies of CT train participants on a variety of popular and widely used tasks divided broadly into the areas of WM, attention, reasoning, planning, visuospatial processing, and verbal processing. Some studies find improvement across a wide array of tasks and cognitive skills, whereas others find more modest effects in only a subset of the outcome measures. In some studies, out of the many comparisons, only a few actually reveal any change or benefit, raising concern for the possibility of a Type 1 error.

## 4. Conclusion

Patients with PD are at an increased risk of cognitive decline. MCI and dementia are significantly more prevalent in PD relative to age-matched controls, and pharmacological treatments for these symptoms are modest at best. Consequently, developing alternative or adjunctive therapies is vital. To date, the small literature investigating CT in patients with PD suggests that these interventions are promising, at least in the immediate or short term for some cognitive domains. However, there remain many unanswered questions. Owing to a lack of consistency across studies in terms of participants included, outcome measures and training interventions selected, and modes of administration with few direct comparisons across alternative groups, regimens, or methods of administration, the efficacy of CT and the expected impact in PD remains largely unknown.

Indeed, it remains unclear if any element(s) in a CT regimen render it effective. The literature is mostly silent on the dosage of intervention required to produce changes and whether any improvements are enduring. There is also a vital need to address the generalizability of CT effects within the framework of transfer of learning. We highly recommend examining transfer of trained skills to practical and functional outcomes that are more similar to daily activities. Examination of QOL changes is also of utmost importance because ultimately the goal is for cognitive improvements to lead to an increased functionality and QOL. Lastly, and most importantly, to advance CT in PD literature, future studies need to provide clear and detailed justification and operationalization of outcome measures and training tasks. Significant changes in outcome measures achieved by training regimens that are rational, theoretically motivated, and hypothesis driven will inspire greatest confidence. Based on the current literature, it is premature to make recommendations for immediate and practical clinical application of CT in PD. This area of research remains in its initial stage but it is crucial that future investigations incorporate clear and appropriate controls, well-described and justified training and outcome tasks, and replications within and between studies.

## Figures and Tables

**Table 1:** Classification of studies of CT in PD according to design.

Single group, uncontrolled studies	Waitlist-controlled studies	Studies comparing CT to standard treatments	Comparing different CT interventions
Sinforiani et al., [68] Mohlman et al., [69] Disbrow et al., [70]	Nombela et al., [71] Naismith et al., [72] Edwards et al., [73]	Sammer et al., [74] París et al., [75] Pompeu et al., [76] Peña et al., [77] Cerasa et al., [78]	Reuter et al., [79] Petrelli et al., [80] Zimmermann et al., [81]

**Table 2:** Summary of studies of CT in PD.

Article by	Participants	Description of training intervention	Outcome measures	Results on outcome measures (# significant differences/total # of measures)	Description of setting # weeks|# sessions|session length (minutes)|total intervention length (hours)	Combined intervention or only CT	Standardized intervention	Assessed QOL
Sinforiani et al., 2004 [68]	20 PD-MCI MMSE~25 No dementia H&Y 1.5	TNP software, focus on attention, abstract reasoning, visuospatial abilities, different level of complexity.	MMSE Digit span Corsi's test COWAT FAS Babcock's story Raven's matrices WCST Stroop test	Pre-post improvement: 3/8 Babcok's story;^*∗*^ COWAT FAS;^*∗∗*^ Raven's matrices^*∗*^	Computerized, hospital program 6|12|60|12	CT and motor rehabilitation	Yes, TNP software.	No

Sammer et al., 2006 [74]	12 PD CT 14 PD standard treatment MMSE~27 No dementia H&Y 2-3	*CT*- BADS (unused subtests); Raven's matrices; picture arrangement tasks, picture completion tasks, block design, object assembly (from WISC); short stories & discussions; pictures prompting stories. *Standard treatment*, occupational therapy, physiotherapy, and physical treatment.	BADS, rule shifting BADS, six elements CET, German version TMT, German version Face name learning test Attention Wellbeing scale Verbal intelligence scale Hamilton Rating Scale for Depression	Pre-post improvement: 2/5 *CT more than standard treatment*, improved on BADS rule shifting^*∗*^ *CT and standard treatment groups*, improved on BADS six elements^*∗∗∗*^	Noncomputerized, hospital program 3-4|10|30|5	Only CT in hospital versus standard treatment	Not standardized intervention. Additionally, task difficulty was adjusted according to each participant's performance level.	Yes. No change (mood questionnaire)

Nombela et al., 2011 [71]	5 PD CT 5 PD untrained 10 healthy controls MMSE 25-26 H&Y 2.5	*PD untrained & healthy controls*, waitlist *CT*, one easy level Sudoku puzzle (4 × 4 grid, 2 × 2 blocks) daily for six months. Weekly meetings.	UPDRS MMSE Stroop accuracy Stroop RT Sudoku RT Brain activation	Posttraining PD CT versus PD untrained: Sudoku, faster solving time^*∗*^ Stroop, more correct answers^*∗*^, fewer missing answers^*∗∗∗*^, lower RT^*∗∗*^. PD CT group showed brain activation pattern more similar to controls.	Noncomputerized, at home with weekly meetings to discuss progress, Sudoku table 1/day, for 6 months Impossible to calculate total training time	Only CT	No, Sudoku plus weekly meetings, much longer duration than traditional CT.	No

Mohlman et al., 2011 [69]	16 PD MMSE 28 No dementia	Attention Process Training II (APT-II), audio CDs, pen and paper worksheets, response clickers. Training sustained attention, divided attention, alternating attention, and selective attention.	Acceptability Feasibility COWAT Stroop Digit span f & b TMT B	Pre-post improvement. No statistics	Computerized + daily practice, in lab, assisted 4|4|90|6	Only CT but not assessing effectiveness	Yes, APT-II.	Not reported

París et al., 2011 [75]	16 PD CT 12 PD control Excluded MMSE <23, some MCI in both groups H&Y 2.37, 2.25	*PD CT*: SmartBrain intervention as well as pen and paper homework. Individualized from a platform of 28 tasks focusing on attention, WM, executive function, memory, visuospatial abilities, psychomotor speed. Also training in language, calculations, and culture. *PD control*: speech therapy, focus on speech and communication difficulties.	MMSE ACE *Attention and WM*: (i) WAIS III Digit Span f & b (ii) CVLT II-List A1 *Information processing speed*: (i) SDMT (ii) TMT A (iii) Stroop, word subtest *Verbal memory*: (i) CVLT-II-Short-Delay Free Recall (ii) CVLT-II-Long-Delay Free Recall (iii) Logical Memory subtest I (iv) Logical Memory subtest II *Learning*: (i) CVLT-II-List A Total *Visual memory*: (i) ROCFT-Immediate Recall (ii) ROCFT-Delayed Recall *Visuoconstructive abilities*: (i) ROCFT-Copy *Visuospatial Abilities*: (i) RBANS-Line Orientation *Verbal fluency*: (i) Phonemic-COWAT FAS (ii) Semantic-COWAT Animals *Executive functions*: (i) TMT-B (ii) TOL-Total Moves (iii) TOL-Total Correct (iv) TOL-Rules Violations (v) Stroop Test-Interference PDQ-39 Mood, geriatric depression scale Cognitive difficulties in activities of daily living, Cognitive Deficits Scale	SmartBrain group improved on 10/23 measures compared to PD control group. *Attention and WM 1/4*: digit span forward^*∗*^ *Information processing speed 1/3*: Stroop word^*∗∗∗*^ *Visual memory 2/4*: ROCFT, immediate^*∗∗*^ and delayed^*∗*^ *Verbal 1/2*: Semantic-Animals^*∗∗*^ but not Phonemic-FAS *Executive functions 3/5*: TMT-B^*∗*^, TOL Total Moves^*∗∗*^, and Total Correct^*∗∗*^	Computerized and noncomputerized plus homework tasks, in lab and at home 4|12|45|9 Plus homework for unspecified amount of time	Only CT versus speech therapy	No, selection of tasks plus SmartBrain, individualized for each participant.	Yes. No change on PDQ39, on measure of mood, or of activities of daily living

Pompeu et al., 2012 [76]	16 PD General balance 16 PD WiiFit H&Y 1-2 MOCA 22-impaired	*WiiFit and cognition* (cognition as part of the game's requirements, not specifically trained). Games used: Single Leg Extension, Torso Twist, Table Tilt, Tilt City, Soccer Heading, Penguin Slide, Rhythm Parade, Obstacle Course, Basic Step, Basic Run. *General Balance * **: **Similar motor requirements as the Wii games.	UPDRS-II (activities of independent living) MOCA Static and dynamic balance measures	WiiFit and general balance exercise groups both showed improvement in UPDRS II^*∗*^(independent activities of daily living scale) and MOCA scores^*∗*^. No difference between groups before, after, or at 60-day follow-up.	Computerized-sessions led by an instructor 7|14|60|14	Combined with global exercises. Computerized but not cognitive focused.	Yes, WiiFit games.	Yes. Both groups improved on UPDRS II-activities of independent living

Reuter et al., 2012 [79]	71 PD CT (group A) 75 PD CT + transfer (group B) 76 CT + transfer + motor (group C) MCI in all groups	*CT*- BADS (unused subtests); Raven's matrices; picture arrangement tasks, picture completion tasks, block design, object assembly (from WISC); short stories & discussions; pictures prompting stories. *CT + transfer*: same as above + daily tasks such as grocery shopping, tending to a vegetable patch, and so forth. *CT + transfer + motor*: same as above + games and tasks to enhance inhibitory control, WM, coordination, and so forth.	ADAS- Cog SCOPA – Cog BADS- six element BADS – zoo map BADS – instruction PASAT Goal Attainment Scale PDQ – 39 UPDRS	No detailed statistics, all groups improved. The more involved groups (groups B and C) improved more. There was a significant group × time interaction, suggesting group C improved more than other groups on ADAS-Cog^*∗∗∗*^ and SCOPA-Cog^*∗∗∗*^	Computerized and noncomputerized, hospital and at home, at least 14 sessions, 4/week, 60 minutes, then at home, 3/week, 45 minutes each. Minimum: 4|16|60|16	Only CT versus CT + transfer training versus CT + transfer training + psychomotor training	No Individualized	Yes. Improvement in order of magnitude C > B > A

Disbrow et al., 2012 [70]	14 PD CT impaired 16 PD CT unimpaired 21 Controls	*Two-phase button press task*, a motor sequence learning task, participants had to press numbered keys corresponding to the number sequence shown on screen. Sequence length varied between 1 and 4 digits.	Motor sequence learning task TIADL TMT D-KEFS TUG	Posttraining, the impaired PD group showed significant improvement in time for sequence initiation, time for sequence completion, and number of errors in the internally represented condition of the task.	Computerized, adaptive difficulty, completed at home 2|10|40|~6.5	Only CT	Yes, but adaptive difficulty.	Yes. No changes in time to complete instrumental activities of daily living

Naismith et al., 2013 [72]	35 PD CT + psychoeducation 15 PD waitlist MMSE 27	*Neuropsychological Educational Approach to Remediation (NEAR)*, individualized, computer based training program devised according to their test results, using a mix of commercially available CT interventions and software programs.	Wechsler Memory Scale III: LOGMEM I - Immediate LOGMEM II – Delayed TMT A TMT B COWAT FAS BDI	CT > waitlist improvement on 2/7 measures: LOGMEM I – Immediate^*∗*^ LOGMEM II – Delayed^*∗*^	Computerized, in lab group sessions 7|14|120|28	CT combined with psychoeducation	No Individualized	Yes No effects on depression BDI.

Edwards et al., 2013 [73]	44 PD Speed of Processing Training (SOPT) 43 PD waitlist H&Y 1–3 MMSE 28	*SOPT*,self-administered, computer based training program that includes 5 exercises aimed at training speed of information processing. The exercises adapt in difficulty according to performance.	UFOV Cognitive Self-Report Questionnaire Depressive symptoms (CES-D)	SOPT > waitlist improvement on 1/3 measures: UFOV^*∗∗*^	Computerized, self-administered, at home 12|36|60| ≥20	Only CT	Standardized program (InSight), individually adaptive difficulty levels.	Yes No effects on depression CES-D

Petrelli et al., 2014 [80]	22 PD NeuroVitalis (NV) 22 PD mentally fit (MF) 21 PD waitlist H&Y 1–3 No dementia MMSE 28	*Structured*: Psychoeducation, group games, individual and group tasks, focusing on attention, memory, and executive functions. *Unstructured*: Group conversation, group games, individual and group tasks, focusing on attention, memory, executive functions, language, and creative thinking. Tasks for each session chosen at random.	DemTect MMSE Brief Test of Attention Memo Complex figure-ROCFT and Taylor COWAT FAS BDI PDQ-39	NV > waitlist improved on 2/12: Memo-Verbal short term attention score^*∗∗∗*^ and DemTect, digit span reverse^*∗*^. MF > waitlist improved on BDI^*∗*^. NV > MF improved on DemTect, digit span reverse^*∗∗*^.	Computerized, pen and paper and activities, in lab group sessions 6|12|90|18	Only CT	NV group standardized intervention. MF unstandardized, unstructured.	Yes. MF improved on BDI scores. No changes in PDQ-39

Zimmermann et al. 2014 [81]	19 PD CogniPlus 20 PD WiiFit MMSE 29 H&Y 1-2	CogniPlus-focused attention; N-Back; planning and action; response inhibition. WiiFit-tennis, swordplay, archery, air sports.	Tests of Attentional Performance-Alertness Tests of Attentional Performance-WM TMT Block design test CVLT	No overall test of improvement for each group separately. WiiFit group improved over CogniPlus group on 1/5 measures: Tests of Attentional Performance-Alertness^*∗*^.	Computerized, in lab supervised by assistant 4|12|40|8	Only CT versus pure Wii sports	Yes, both interventions.	No

Peña et al., 2014 [77]	22 PD REHACOP 22 PD occupational therapy MMSE 27	REHACOP, group sessions including focus on attention, memory (visual and verbal, recall and recognition), language and verbal processing, executive functions (planning and logical reasoning), social cognition and Theory of Mind.	*Processing speed*: TMT A Salthouse letter comparison test *Verbal memory*: Hopkins verbal learning test, learning and long term recall *Visual memory*: Brief visual memory test, learning and long term recall *Executive function*: Stroop word color, interference scores Theory of Mind: Happé test	REHACOP > occupational therapy improved on 4/9 measures. Processing speed^*∗*^ Visual memory^*∗*^ Theory of Mind^*∗*^ Functional disability^*∗*^	Noncomputerized, psychologist led group sessions 13|39|60|39	Only CT	Yes, REHACOP modules.	Yes. Functional disability scores improved in REHACOP group more than occupational therapy group

Cerasa et al., 2014 [78]	8 PD RehaCom 7 PD coordinated tapping task	RehaCom, computer assisted training of attention and information processing. Tapping task, also computerized, using in-house software.	ROCFT Selective Reminding Test Judgement Line Orientation COWAT SDMT PASAT Digit span f & b Stroop TMT A & B	RehaCom > control tapping group improved on 2/20 measures. Digit span forward^*∗*^ SDMT^*∗∗*^	Computerized, group sessions with weekly meetings 6|12|60|12	Only CT	Yes, RehaCom training.	Yes. No changes in PDQ-39 scores or measures of mood

*P* value indicators:

*∗*: <0.05.

*∗∗*: <0.01.

*∗∗∗*: <0.001.

## Abbreviations

ACE: Addenbrooke Cognitive Examination
ADAS-Cog: Alzheimer's assessment scale
BADS: Behavioral assessment of the dysexecutive syndrome
BDI: Beck Depression Inventory
CES-D: Centre for Epidemiological Studies-Depression Scale
CET: Cognitive estimation test
COWAT: Controlled Oral Word Association Test
CT: Cognitive training
CVLT: California Verbal Learning Test
D-KEFS: Denis-Kaplan Executive Function Scale
f & b: Forward and backward
H&Y: Hoehn and Yahr Scale
MCI: Mild cognitive impairment
MF: Mentally fit
MMSE: Mini-Mental State Examination
MOCA: Montreal cognitive assessment
NV: NeuroVitalis
PASAT: Paced auditory serial attention test
PD: Parkinson's disease
PDQ-39: Parkinson's disease questionnaire
RBANS: Repeatable battery for the assessment of neuropsychological status
ROCFT: Rey Osterrieth complex figure test
RT: Reaction time
SCOPA-Cog: Scales for outcome of Parkinson's disease
SDMT: Symbol digit modality test
SOPT: Speed of Processing Training
TIADL: Timed instrumental activities of daily living
TMT: Trail Making Test
TNP: Training
TOL: Tower of London
TUG: Timed-Up-and-Go Test
UFOV: Useful field of view
UPDRS: Unified Parkinson's disease rating scale
WAIS: Weschler adult intelligence scale
WCST: Wisconsin card sorting task
WISC: Wechsler intelligence scale-children's version
WM: Working memory.
